# Liver Cirrhosis: The Immunocompromised State

**DOI:** 10.3390/jcm13185582

**Published:** 2024-09-20

**Authors:** Elda Victoria Rodríguez-Negrete, Marisol Gálvez-Martínez, Karina Sánchez-Reyes, Carlos Fernando Fajardo-Felix, Karla Erika Pérez-Reséndiz, Eduardo Osiris Madrigal-Santillán, Ángel Morales-González, José Antonio Morales-González

**Affiliations:** 1 Servicio de Gastroenterología, Hospital de Especialidades, Centro Médico Nacional Siglo XXI, Ciudad de México 06720, Mexico; jev_rn@yahoo.com.mx (E.V.R.-N.); marygama84@hotmail.com (M.G.-M.); fajardocf92@gmail.com (C.F.F.-F.); karlaerikapr@gmail.com (K.E.P.-R.); 2 Laboratorio de Medicina de Conservación, Escuela Superior de Medicina, Instituto Politécnico Nacional, Mexico City 11340, Mexico; eomsmx@yahoo.com.mx; 3 Servicio de Cirugía General, Hospital de Especialidades, Centro Médico Nacional Siglo XXI, Ciudad de México 06720, Mexico; drakarinacg@yahoo.com.mx; 4 Escuela Superior de Cómputo, Instituto Politécnico Nacional, Unidad Profesional “A. López Mateos”, Ciudad de México 07738, Mexico

**Keywords:** liver cirrhosis, cirrhosis-associated immune dysfunction, systemic inflammation, bacterial translocation

## Abstract

Systemic inflammation and immunodeficiency are important components of cirrhosis-associated immune dysfunction (CAID), the severity of which is dynamic, progressive, and associated with the greater deterioration of liver function. Two inflammation phenotypes have been described: low-grade and high-grade systemic inflammation. Both of these phenotypes are related to liver cirrhosis function; thus, high-grade inflammation is correlated with the severity of hepatic insufficiency, bacterial translocation, and organic insufficiency, with which the risk of infections increases and the prognosis worsens. Bacterial translocation (BT) plays a relevant role in persistent systemic inflammation in patients with cirrhosis, and the prophylactic employment of antibiotics is useful for reducing events of infection and mortality.

## 1. Introduction

Liver cirrhosis is a chronic disease (because it generates complications such as portal hypertension and the development of hepatocellular carcinoma), and it is associated with frequent hospitalizations and high mortality; it represents 1.2 million deaths annually worldwide and is the 14th leading cause of death worldwide [1].

Cirrhosis is classified into two stages: compensated cirrhosis and decompensated cirrhosis. Acute decompensation (AD) involves the development of an acute complication (e.g., ascites, variceal hemorrhage, hepatic encephalopathy), and the first event of AD marks the transition from compensated to decompensated cirrhosis. The evolution of decompensated cirrhosis is characterized by repeated episodes of AD in which patients are prone to developing bacterial infections [2]. Finally, Acute-on-Chronic Liver Failure (ACLF) is the presence of insufficiency of an organic system or multiple organic systems, with high short-term mortality, and always occurs within the context of AD. Systemic inflammation (SI) is a well-recognized characteristic of decompensated cirrhosis [2]. Cirrhosis affects various organs and systems, including the immune system [3], is considered an immunocompromised state that increases the risk of infections, and has an approximate mortality of 30% [4]. The term cirrhosis-associated immune dysfunction (CAID) is characterized by two important components: immunodeficiency due to an altered response to pathogens and systemic inflammation as a consequence of a persistent and inadequate stimulation of the immune system. CAID should be considered a complication of cirrhosis of any etiology [5], a multifactorial state that diminishes the patient’s capacity to eliminate bacteria, cytokines, and endotoxins from the circulation [6].

### 1.1. Immunological Role of Resident Cells in the Liver

The liver is a component of the innate immunological system, which contains 90% of the body’s reticuloendothelial (RE) cells [7,8]. Within the liver, antigen-presenting cells (APCs); T and B cells; natural killer (NK) cells; and finally monocytes control the local and systemic inflammatory response and, thus, are fundamental for the elimination of bacteria. Hepatocytes serve as APCs because they show major histocompatibility complex (MHC) type I and II [9]. In a sterile injury, the release of DAMPs from necrotic cells triggers the recruitment of innate immune cells [8,10]. In healthy patients, during an acute phase or systemic inflammatory response, a variety of pro-inflammatory cytokines (IL-6, IL-1, TNF, and IFN-γ) can stimulate hepatocytes to produce high levels of complements and PRRs (recognition receptors of patterns); however, this is altered in patients with liver cirrhosis [8,11].

Progressive damage to liver tissue (parenchymal and non-parenchymal cells) decreases the activity of the mononuclear phagocytic system, leading to an increased risk of infections and mortality [3,12]. Furthermore, intrahepatic shunts through vascularized septa prevent systemic and portal bacteria from being filtered and eliminated by Kupffer cells [13]. Associated with this is the loss of hepatocytes, which diminishes the synthesis of proteins (albumin and acute phase protein) and immune receptors (complement components, soluble PRRs) [14]. The serum concentrations of C3 and C4, as well as the hemolytic activity of the complement, are reduced in patients with decompensated cirrhosis compared to controls; these alterations predispose to infectious processes and confer higher mortality in patients with alcoholic cirrhosis [15].

### 1.2. Systemic Inflammation in Cirrhosis

**Figure 1:**
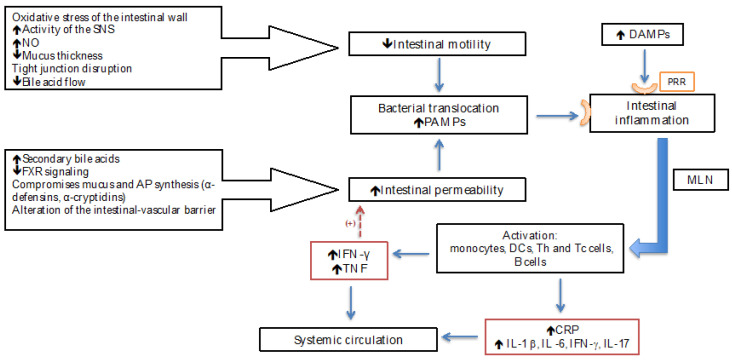
Changes in intestinal motility and permeability in liver cirrhosis contribute to the promotion of bacterial translocation and increased presence of PAMPs. Concurrently, DAMPs are released from necrotic cells, and both PAMPs and DAMPs are recognized by PRRs, triggering intestinal inflammation and subsequently leading to systemic inflammation due to the release of pro-inflammatory cytokines and acute phase proteins. PAMPs: pathogen-associated molecular patterns; DAMPs: danger-associated molecular patterns; PRRs: pattern-recognition receptors; SNS: Sympathetic Nervous System; FXR: farnesoid X receptor; APs: antimicrobial peptides; DCs: dendritic cells; MLNs: mesenteric lymph nodes; CRP: C-Reactive Protein; NO: nitric oxide.

Pathogen-associated molecular patterns (PAMPs) are molecules derived from bacteria, such as lipopolysaccharides (LPSs) from Gram-negative bacteria, lipoteichoic acid from Gram-positive bacteria, and DNA regions, as well as viruses. Danger-associated molecular patterns (DAMPs) are molecules found inside cells and released by damaged cells [3,8]. Inflammation is a physiological response that restores homeostasis after multiple aggressions, for example, bacterial infections or tissue lesions; similarly, it may originate from recognizing distinctive bacteria-derived molecules (LPSs), described as PAMPs [3,16]. PAMPs and DAMPs bind with PRRs (pattern-recognition receptors) that are expressed in epithelial and peripheral innate immune cells. PRRs can be localized on the cellular surface (the type-4 Toll-like [TLR4]), the endolysosome (TLR9), or the cytosol (Nucleotide Oligomerization Dominion [NOD] 1) [3,17]. Once DAMPs and PAMPs reach the liver, they are recognized by PRRs, and pro-inflammatory signaling pathways are activated that aim to restore homeostasis through the release of pro-inflammatory cytokines (TNF; IL-1 β; IL-6; IFN-γ; IL-17; monocyte chemotactic protein type 1; macrophage inflammatory protein beta; the activation of neutrophils; monocytes; T lymphocytes; and the activation of endothelial molecules such as cell-adhesion molecule type 1 (ICAM-1), vascular adhesion type 1 (VCAM 1), endothelial growth factor (VEGF), Von Willebrand factor (vWF), and nitrates) and C-Reactive Protein (CRP) [3,18]. PAMPs in the intestinal lumen are recognized by PRRs in gut-associated lymphoid tissue (GALT) and mesenteric lymph nodes. The latter leads to gene expression through activating immune cells to produce molecules responsible for intestinal inflammation and finally extends to the systemic circulation and the peripheral organs [19]. The PAMP-induced inflammatory response is indispensable for combating invasive bacteria; however, an excessive or chronic response can cause collateral tissue damage (immunopathology) and provoke a marked compensatory anti-inflammatory response that, in the latter instance, leads to immune suppression and a greater risk of secondary infections and, with that, greater mortality [20] (Figure 1).

### 1.3. Evidence of Systemic Inflammation

Cirrhosis is associated with SI, as evidenced by increased white blood cell count, neutrophils, activated circulating monocytes, plasma CRP, pro-inflammatory cytokines, macrophage activation markers, and systemic oxidative stress [17].

As liver function deteriorates, systemic inflammation increases; this is characteristic of CAID [3]. Short-term mortality (3 months) in ACLF has been associated with the activation of mediators of leukocyte adhesion and migration (VCAM1, ICAM1, granulocyte, and macrophage colony-stimulating factor [GM-CSF]) [21]. 

#### 1.3.1. Low-Grade Systemic Inflammation

BT is defined as the passage of viable bacteria or bacterial subproducts through the intestinal mucosa to the systemic circulation [2]. In cirrhosis, intestinal permeability increases due to the modification of the mucosal lining, the intercellular binding proteins, the loss of epithelial tight junction proteins through TNF receptor-I-mediated activation of myosin light-chain kinase, and the endothelial cells. In addition, the excessive growth of bacteria and dysbiosis favors the arrival of bacteria and their products into the systemic and portal circulation [22,23].

In healthy patients, gut bacteria reside in a symbiotic state along with the host; dysbiosis is the quantitative and/or qualitative change in the microbiota and occurs due to the modification of intestinal homeostasis [17]. Liver cirrhosis is associated with both an overgrowth of intestinal bacteria and dysbiosis, in the sense that native bacteria decrease and potentially pathogenic bacteria increase; for example, there are fewer Bacteroidetes but more Proteobacteria and Fusobacteria [24]. Another common intestinal characteristic of cirrhosis is an altered intestinal barrier, which leads to pathological BT [25]. Hypergammaglobulinemia, which is common in decompensated cirrhosis, is a sign of escape of intestinal antigens into the systemic circulation and correlates with clinical outcomes [2]. BT and SI are continually present in patients who are non-infected with decompensated cirrhosis [2,26].

Infections increase mortality by four times among patients with cirrhosis; 30% of cases occur within the first month after the infectious process, and 63% of patients die within 1 year. The Odds Ratio (OR) for dying in infected vs. non-infected patients is 3.75 (95% Confidence Interval [CI]: 2.12–4.23) [27]. Patients with cirrhosis who are hospitalized exhibit a greater risk of becoming infected, particularly those with gastrointestinal (GI) bleeding. A 2001 study identified 34% (*n* = 150) of bacterial infections in patients admitted with cirrhosis (89 were community-acquired, and 61 were hospital-acquired), where the urinary tract was the most frequent site of infection (41%); 60% of cases at the time of admission already had an infectious process, and 40% occurred during hospitalization, where the prevalence of bacterial peritonitis was 12%. However, it is noteworthy that 51% of bacterial peritonitis was found in asymptomatic patients; the main bacteria found were Gram-negative [28].

Since 1996, the effectiveness of antibiotic treatment has been evaluated in preventing bacterial infections after hemorrhage in patients with cirrhosis. In one study, three groups were assigned: group 1 included patients with Child–Pugh A or B without bleeding (without antibiotic prophylaxis); group 2 included patients with Child–Pugh A or B and bleeding events, as well as patients with Child–Pugh C with hemorrhage without antibiotic prophylaxis; and group 3 included patients with Child–Pugh C with hemorrhage and antibiotic prophylaxis for 10 days. The incidence of bacterial infection was significantly higher in patients in group 2 than in those of group 1, and infections were more severe in group 2; however, when comparing mortality between groups 2 (23.5%) and 3 (13.3%) during the first 4 weeks, there was no significant difference [29].

In the PREDICT study, 1071 patients with AD were included; three groups of patients were identified, with a differential white blood cell count and CRP levels (as markers of inflammation); patients with pre-ACLF (*n* = 218) and who developed ACLF during the first hospitalization (20%) had a mortality rate of 53.7% at 3 months and 67.4% at 1 year. Patients with unstable decompensated cirrhosis (*n*= 233) and who required hospitalizations on several occasions without developing ACLF had lower mortality rates at 3 months (21%) and at 1 year (35.6%). Patients with stable decompensated cirrhosis (*n* = 620) who did not require hospitalization or develop ACLF had a 1-year mortality rate of 9.5% [30].

#### 1.3.2. High-Grade Systemic Inflammation

Bacterial infections are the most common trigger of ACLF [6]. The chronic consumption of alcohol alters the intestinal barrier, promoting dysbiosis, altering the tight junctions, decreasing the mucous thickness, reducing intestinal farnesoid X receptor (FXR), and diminishing the production of antimicrobial peptides (which play a crucial role in the innate immunity of the host against a broad range of microorganisms). In ACLF, there is an increase in BT [31,32]. Alcohol alters innate and adaptive responses; similarly, dietary factors (fructose, saturated fats, trans fats, and cholesterol) trigger inflammation via lipotoxic effects, alterations in mitochondrial function, and oxidative and endoplasmic reticulum stress [33].

### 1.4. Consequences of Systemic Inflammation

Impaired peripheral, portal, and systemic vasodilatation is caused by SI and the endothelial production of nitric oxide (NO) and reactive oxygen species (ROS). Cardiac dysfunction is related to the severity of systemic circulatory dysfunction, as well as markers of SI and BT [3,34,35]. Patients with AD present persistent low-grade inflammation, with the deterioration of portal pressure and, in turn, increased 1-year mortality (35%); those with pre-ACLF exhibit a high-grade inflammation, which gives them a mortality of 67.4% [3]; with the progression of liver disease, the biomarkers of systemic inflammation increase. High-grade SI is manifested by organ failure due to tissue damage (immunopathology) or an energy imbalance (immunometabolism). In ACLF, the risk of acute renal failure increases due to microthrombosis, capillary leukocyte infiltration, and mitochondrial injury, leading to the development of ischemic acute renal tubular necrosis [3,36,37].

The degree of inflammation parallels the presence of hepatic encephalopathy and the severity of hepatic, circulatory, and renal dysfunction [6,19,38].

### 1.5. Immunodeficiency and Cirrhosis

The abnormalities of immune system cells compromise their effector function and cause immune paralysis (cellular immune depression), including functional defects (production of TNF-α and a diminished HLA-DR expression on monocytes), the expansion of immune inhibitors, and/or the reduced expression of costimulatory molecules (IFNγ and TNF) [39]. Immunodeficiency is a feature of CAID, present in compensated cirrhosis, but is further altered in decompensated cirrhosis, reaching its maximal point in ACLF [40].

### 1.6. Progressive Loss of Tolerance in Cirrhosis

A progressive deterioration of tolerance to antigen recognition in cirrhosis allows an increase in the pro-inflammatory response and contributes to chronic inflammation. In patients with compensated cirrhosis, monocytes increase their expression of HLA-DR and increase the spontaneous production of TNF (due to the alteration in the inducible suppressor of TNF) and IL-10; however, when the disease progresses—for example, patients with ACLF and acute decompensation of cirrhosis had increased numbers of MER receptor tyrosine kynase (MERKT)-expressing monocytes and macrophages—this results in a decrease in the activation of TLR and pro-inflammatory cytokines and an increase in anti-inflammatory cytokines (IL-10). MERKT negatively controls the innate immune response. In ACLF, MERKT expression was correlated with the severity of hepatic and extrahepatic disease and the systemic inflammatory response [13,41].

### 1.7. Damage to Circulating Immune Cells

Immune dysfunction in cirrhosis affects the innate and adaptive immune cells, showing cellular hyperactivation and altered effector response [3] (Table 1).

Immunodeficiency is manifested by alterations in APCs (i.e., monocytes and neutrophils) and reduced phagocytic capacity; however, it is more pronounced in patients with a greater deterioration of liver function. Immunodeficiency in ACLF is due to a diminished HLA-DR expression and damage to TNF production as a consequence of LPS by monocytes and elevated levels of MERKT (receptor tyrosine kinase-expressing monocytes and macrophages), which produce IL-10 [42].

### 1.8. Compensatory Anti-Inflammatory Response Syndrome (CARS)

Decreased effector activity by lymphocytes against an antigen, a decrease in cytokine production, HLA expression after monocytes stimulation, and an upregulation of anti-inflammatory cytokines define compensatory anti-inflammatory response syndrome (CARS) [43]. In a cohort of 51 patients with a diagnosis of ACLF and decompensated cirrhosis, the degree of SI (levels of IL-10, IL-6, and TNF-α) determines the outcome. In patients with ACLF, IL-10 levels are higher and correlated with a poor prognosis. In addition to IL-10, another finding is that together with the International Normalized Ratio (INR) (OR 1.7), at the moment of admission, they predict unfavorable clinical outcomes [44]. IL-10 constitutes an important part of CARS, which is closely related to the functional deactivation of monocytes, immunoparesis, and predisposition to recurrent and opportunistic infections [45].

### 1.9. Intestinal–Liver Axis and the Intestinal Immune System

The intestinal–liver axis plays an important role in the development of CAID [3]. To maintain tolerance to harmless stimuli, interactions of the vascular barrier, intestinal epithelium, microbiota, liver, and immune system are important. Cirrhosis presents dysbiosis and increased intestinal permeability that promote BT, notably contributing to SI [3,46].

Intestinal macrophages kill the translocated bacteria; intestinal dendritic cells (DCs) transport them alive to mesenteric lymph nodes to stimulate the adaptive T-cell response; 94% of CD4 T cells are found in the GALT. Intraepithelial lymphocytes, particularly the γδ subpopulation, maintain a close relationship with intestinal epithelial cells, acting as essential mediators that balance host microbial homeostasis [4,46,47].

Intestinal motility is decreased in patients with cirrhosis, which favors overgrowth within the intestine. This, together with the portosystemic shunt, allows the perpetuation of the bacteria and can cause bacteremia [48]. This, in turn, causes greater oxidative damage due to the increase in endotoxins, pro-inflammatory cytokines, and NO [49]. Peptidoglycans, or LPSs, bind to Toll-like receptors, initiating the cellular signaling cascade and releasing cytokines (TNF-α, IL-6, and IL-1). In systemic inflammatory response syndrome (SIRS), anti-inflammatory cytokines (IL-10, IL-4, IL-13, and PGE2) balance pro-inflammatory cytokines, a process known as the “cytokine storm”, leading to excessive inflammation [50]. The development of SIRS is determined by the severity of the liver disease; this, in turn, favors the presence of variceal hemorrhage and hepatic encephalopathy and negatively affects survival [51].

In experimental models of cirrhosis, intestinal CD103^+^ DCs are activated when the decompensation of liver function (ascites) occurs, and there is an expansion of pro-inflammatory CD4^+^ DCs, which favors the increase in the production of TNF and the increase in phagocytosis and migration capacity [52]. When ascites develop, T helper cells switch to T_H_1, thus increasing TNF and IFNγ production in the lamina propria, with a concomitant T_H_17 depletion and an increase in IL-1 and IL-18 in ascites but not in serum [35,53].

In spontaneous bacterial peritonitis (SBP), there is a difference between the low microbial load in ascites and the intensity of the inflammatory response in the peritoneum, which shows a correlation between SI and prognosis [54]. During SBP, CD206 levels in ascites, but not in serum, correlate with SI, peritoneal inflammation, and mortality; the massive release of pro-inflammatory cytokines during bacterial infection contributes to SI and organ failure [55].

The use of non-absorbable antibiotics decreases NO production, inflammation, and hemodynamic alterations [56,57] and reduces endotoxemia and the severity of liver disease [3].

### 1.10. Humoral Factors in Circulation

Norepinephrine acts on the microbiome, has harmful effects on enterocytes, increases intestinal permeability, and facilitates the perpetuation of intestinal inflammation. In ACLF, norepinephrine levels are three times higher in AD and strongly correlate with the severity of the systemic inflammatory response [58,59].

The adaptive immune response is induced and regulated by T helper (Th) cells. Patients with cirrhosis frequently present with Th cell lymphopenia due to splenic sequestration, as well as a reduction in the naïve Th cell compartment [60]. Lario et al. examined circulating lymphocytes, determining their number and distribution; compared with controls, they showed a drastic reduction in the number of Th and cytotoxic T (Tc) cells. The number of naive T cells was reduced 2.7-fold, while the number of memory Th cells was reduced 1.5-fold in patients with cirrhosis. The reduction in naïve Th cells favors the state of lymphopenia in cirrhosis, characterized by an increase in memory Th cells, increased apoptosis, and the accumulation of splanchnic Th cells [61].

In cirrhosis, endothelial activation and hemodynamic changes are related in part to elevated levels of TNF-α [62]. The plasma levels of LBP increase in response to LPSs. Its function is to transport LPSs to effector immune cells, and the decontamination of the intestine with norfloxacin decreases the risk of bacterial infections, decreases circulating levels of TNF-α and LBP, and improves the hemodynamic status of patients with advanced cirrhosis [63].

A prospective study included 25 healthy subjects and 60 patients with alcoholic cirrhosis with at least 1 year of abstinence from alcohol; the patients were aged between 25 and 70 years, and ascites were detected in 28/60 patients (46%). Serum LBP levels were above the threshold level of healthy controls in 11 of the 28 patients with ascites; peripheral blood had a significant increase in the total number of monocytes in patients with ascites compared to patients without ascites, in healthy patients, in patients with ascites with normal LBP or without ascites, and healthy controls. The levels of LPS, LBP, sCD14, TNF-α, sTNFα-RI, and IL-6 were increased; it was evident that the behavior of T cells was depleted and that enteric bacteria play a relevant role in these abnormalities of cellular immunity [35].

The subtype of patients with cirrhosis and ascites revealed a higher number of monocytes and a higher expression of HLA-DR on the cell surface and, in particular, of sCD14 and CD80 molecules due to LPS signaling [64].

There was reduced expression of HLA-DR antigen-presenting molecules in patients with AD, resulting in decreased monocyte activation and, in turn, cytokine secretion. In addition to the dysfunction of the RE system, patients with cirrhosis demonstrate a diminution of neutrophil mobilization and phagocytic activity, a phenomenon that correlates with the severity of liver disease [65]. Immunoglobin levels (IgM, IgG, and IgA) are decreased in the ascites of patients with cirrhosis, and the concentrations of C3, C4, and CH50 are significantly lower in serum as well as in ascites [66].

Most bacteria that cause serious infections must be phagocyted and eliminated by phagocytic cells. This process requires that the surface of the bacterial cell be first opsonized with IgG and/or with the third component of this process that requires complement (C3), with the fixation of the complement to the surface of the bacteria being the most important step in opsonization. Complement deficiencies are known to predispose individuals to bacterial infections [67,68]. Bruce et al. determined how opsonization activity in ascites correlates with the content of total protein (CH_100_) and complement (C3 and C4) in ascites; CH_100_ was measured in both ascites as well as in serum, observing that ascites caused by cirrhosis present less opsonic activity than in ascites of other etiologies [66].

There is a correlation between the presence of liver failure and elevated circulating levels of endotoxins, and plasma levels of endotoxins and serum bilirubin are important factors for predicting short-term survival [69]. In cirrhosis, there is a reduction in anti-inflammatory and antiapoptotic factors, high-density lipoproteins, and Protein C, but NO is increased; NO contributes to oxidative stress and causes greater alteration in vasodilation because endotoxemia improves the expression of iNOS [70].

Patients with cirrhosis present alterations in immune function that mainly involve components of the innate immune system. This system represents the first line of defense against pathogens [71]. B cells are part of the humoral immune system, and they are responsible for protection against pathogens and contribute to immune regulation and the maintenance of self-tolerance [71]. B cells are activated as a consequence of the activation of T cells; they migrate to the germinal center where they proliferate and differentiate into memory B cells and antibody-secreting cells; there is an increase in the secretion of immunoglobulins, which forms immune complexes and further activates the immune response leading to liver injury; B lymphocytes are cells that produce pro- or anti-inflammatory cytokines and regulate the immune response [5].

Between 60 and 70% of circulating B lymphocytes are naïve B cells, which express immunoglobulins D and M [72]; when naïve B cells recognize antigens within the germinal centers, memory B cells (MBCs) develop, which return to the peripheral blood. Approximately 96% of MBCs are CD27+ and are B cells that have been exposed to protein antigens. Plasmablasts represent 3% of circulating B cells and are the last stage of differentiation. They are a product of MBCs, and in the bone marrow, they complete their maturation and finally become plasma cells [72,73].

Alcohol consumption damages hepatocytes and leukocytes due to the formation of ROS and the production of acetaldehyde (a breakdown product of alcohol), which destroys cell membranes [71]. Patients with alcohol liver disease (ALD) have an altered B cell compartment, manifested by a significant reduction in memory and naïve B cells (which is associated with increased susceptibility to infection and poor response to vaccination) [74,75], while the percentage of plasma cells is elevated. This increase in plasma cells may be responsible for high levels of IgA, IgG, and IgM in ALD. In Cardoso’s study, it was shown that these cells have a greater increase in acute decompensation than in stable disease [76].

### 1.11. Infections in Cirrhosis

GI bleeding confers a higher risk of infection; about 17% to 45% of patients develop SBP or bacteremia [70]. The development of infection increases the risk of early bleeding; therefore, the preferred prophylactic strategies are third-generation cephalosporins, both for Gram-negative and Gram-positive bacteria. It has been shown that intravenous treatment with 1 g of ceftriaxone for 7 days after the bleeding event is more effective in preventing bacterial infections than oral antibiotics in patients with advanced cirrhosis [77]. Prokinetic agents can reduce dysmotility and BT; prophylaxis with antibiotics and prokinetic agents reduces the risk of SBP compared with antibiotics alone [78].

The prevalence of SBP in hospitalized patients with cirrhosis and ascites ranges from 10% to 30%, with approximately half of the cases occurring at the time of hospitalization and the other half of cases occurring during hospitalization [79]. The hospital mortality rate due to SBP is approximately 32%; renal failure develops in approximately one-third of all patients with SBP and is a strong predictor of mortality during hospitalization [80]. The activation of the cytokine cascade and the production of NO in cirrhosis and SBP negatively impacts kidney function; therefore, the use of intravenous albumin (1.5 g/kg within 6 h of SBP followed by 1 g/kg on day 3) in conjunction with cefotaxime reduces the incidence of kidney failure from 33% to 10% and the incidence of mortality from 29% to 10%. The mean arterial volume improves with albumin, as it binds to TNF-α and NO to compensate for the inflammatory state due to the infectious process, eliminating toxins from the circulation [81].

In patients without antibiotic prophylaxis, the SBP recurrence rate was 43% at 6 months, 69% at 1 year, and 74% at 2 years after the initial episode. Ginés et al. determined that 400 mg of norfloxacin orally per day reduced the recurrence of SBP from 68% to 20% [82].

It has been shown that in-hospital mortality is similar between patients with severe sepsis (32%) and ACLF (30%). The determination of CRP and procalcitonin (PCT) is higher in the sepsis group compared with patients with ACLF; the serum concentrations of IL-6 and IL-10 are higher in severe sepsis and different from those in subjects with ACLF or with stable cirrhosis. In contrast, CRP, PCT, and the average levels of IL-6 and IL-10 are higher in ACLF than in patients with decompensated cirrhosis [39].

As previously described, SI occurs in a setting attributable to the translocation of pro-inflammatory signals from the intestinal lumen to the systemic circulation and/or the release of DAMPs that trigger pro-inflammatory mediators [16,83]. The direct deleterious effects of these pro-inflammatory mediators on organ microcirculation and the homeostasis of cellular physiology can lead to organ failure [10]. At any stage of cirrhosis, patients can develop AD, but patients will only develop ACLF when systemic inflammation is widely activated [84].

The triggers of ACLF are not fully understood; the SI may be increased and allow the development of ACLF without an external trigger [85].

Monteiro et al. evaluated inflammasome activation, estimated by the pro-inflammatory cytokines IL-1α and IL-1β of compensated and recompensated patients, and its role in developing fatal ACLF. The hypothesis of this study was that SI is a prerequisite and necessary for the development of ACLF in patients with compensated and recompensated states. In total, 88 (35%) patients died; the most common cause of death was ACLF in 52 (58.5%) patients. In 63.5% of cases, the trigger of ACLF was not identified. The recompensated patients had significantly higher rates of fatal ACLF and overall mortality compared to those with compensated patients. IL-1β levels were higher and detectable in patients with ACLF compared to those without ACLF (69% vs. 34%); patients with ACLF demonstrated significantly higher rates of IL-10 and IL-1β compared to patients without ACLF [86].

In the observations by Rolando et al., patients with acute hepatic failure (*n* = 887) were included; 56% of them presented SI regardless of whether or not they had bacterial infections. The prevalence of infections in patients with gastrointestinal hemorrhage is less than 2%. SI favors the development of ascites and kidney damage; in 30% of hospitalized patients with AD, bacterial infections and ascites coincide. SI can increase portal hypertension, and its severity correlates with the severity of portal hypertension in patients with cirrhosis. SI activates TLR on HSCs, causing them to become activated and increasing the production of pro-inflammatory cytokines and ROS; however, SI can generate an imbalance within the liver between vasoconstrictor and vasodilator mechanisms, resulting in increased vascular resistance [87].

### 1.12. SI Generates Complications and Organ Failure in AD

Two studies, CANONIC and PREDICT, described ACLF at hospital admission (CANONIC study) and critical periods before and within 3 months after admission in AD non-ACLF (PREDICT study). Mortality rates increased progressively and in parallel with the severity of AD at 3 months and 1 year after admission [6,30,88].

“Sterile” inflammation can derive from acute hepatic inflammatory processes [89]. The preactivation of the innate immune system has been observed to be induced by an exaggerated inflammatory response to bacterial infections and other pro-inflammatory stimuli; the inflammasome is highly active in ascites in patients with cirrhosis without the presence of infection, explaining the exacerbated inflammatory response in the inflammasomes that is associated with a higher degree of liver disease [53].

IL-6 is a sensitive marker of SI; among the 1211 patients with AD, the plasma level of IL-6 was normal upon admission in 3.3% (*n* = 40) of patients; 37 of them exhibited elevated plasma levels of other SI markers (TNF-α, IL-8, IL-10, IL-1RA, and CRP). Of the patients with compensated cirrhosis without a history of AD (*n* = 97), 48 (49.4%) showed normal plasma levels of IL-24 and IL-6. The SI is a prognostic marker in AD and correlates with the number of decompensations [18,30].

Patients with any precipitating factor represent 44% of patients with AD non-ACLF and 70% of AD-ACLF patients [2]. BT is probably the precipitant of SI and AD in 30% of patients with ACLF-AD. The number of precipitants influences the severity of SI [88].

Traditionally, it has been thought that SI causes organ failure due to two different mechanisms that can occur at the same time; SI stimulates the production of NO, causing greater damage to the systemic circulation, which results in increased inflammation, decreased effective arterial blood volume, and the overactivation of endogenous vasoconstrictor systems, resulting in organ hypoperfusion and the subsequent impairment of organ function. SI activates immune cells, resulting in tissue injury and organ damage [17]. It has been suggested to involve metabolic alterations associated with SI, which condition the development of organic dysfunction in cirrhosis [90].

### 1.13. Immunoparesis

Immunoparesis is a mechanism that alters the response of the immune system and thus increases the risk of patients acquiring infections; this term was initially used in patients with sepsis [91]. In a study by J. Fernández et al., 407 patients with ACLF were included, as well as 235 patients with AD. A total of 152 patients (37%) had a bacterial infection at the diagnosis of ACLF; 117 patients (46%) of the remaining 255 patients with ACLF developed a bacterial infection during the 4-week follow-up. Serious infections (SBP, pneumonia, severe sepsis/shock, and nosocomial infections) were more frequent in ACLF. Patients with ACLF and bacterial infections (either at diagnosis or during follow-up) showed a higher degree of SI at diagnosis, a worse prognosis, and decreased 90-day survival compared to patients with ACLF without infection. It is more common to develop fungal infections during the ACLF event than in AD, and this infection gives patients a high 90-day mortality (71%) [92]. In the PREDICT study, the reported incidence of infections in patients with AD non-ACLF was 53% [88,93]. In decompensated cirrhosis, neutrophils show alterations in migration, bacterial identification, and phagocytosis, as well as an increase in ROS production [94,95].

### 1.14. Dysfunction of Immune Cells Due to Metabolic Anomalies of Cirrhosis

In healthy individuals, albumin can bind to pro-inflammatory molecules in the serum and immunosuppressive mediators (PGE), whose serum concentrations are elevated in cirrhosis. In addition to presenting low albumin concentrations, it alters their immune function [96]. Low serum albumin levels (<30 mg/dL) are one of the causes of immunosuppression in acute decompensation and end-stage liver disease. They present elevated concentrations of circulating PGE2 secondary to increased production, which, in combination with hypoalbuminemia, drives innate immune dysfunction, resulting in vulnerability to infection. Human albumin infusion reduces the circulating levels of PGE2 and attenuates the suppressed secretion of pro-inflammatory cytokines from macrophages [58].

### 1.15. Sarcopenia in Cirrhosis

**Figure 2:**
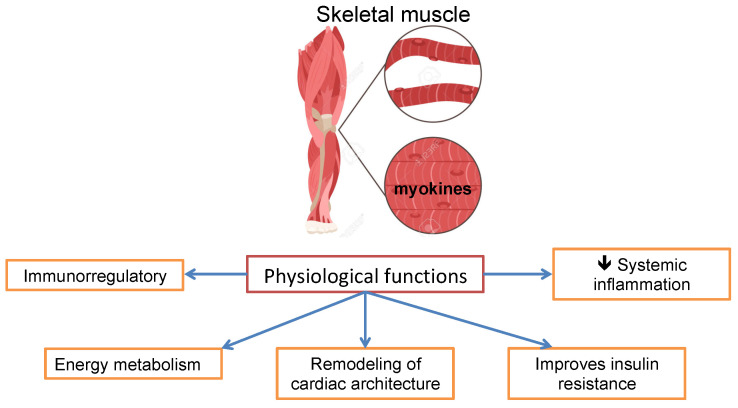
Physiological functions of skeletal muscle.

The progressive and widespread loss of skeletal muscle, strength, and function is defined as sarcopenia, which is observed in up to 70% of patients with cirrhosis. It is relevant as it leads to physical disability and functional impairment, low quality of life, poor prognosis, and increased mortality before and after liver transplantation [97,98]. Its prevalence is high in patients with cirrhosis and low left ventricle mass due to decreased (Neuregulin/Epidermal growth factor) NRG/ERB signaling; this causes a decrease in myogenesis and myoblast differentiation [99]. Skeletal muscle releases myokines upon contraction and degradation. This is relevant because these processes have multiple beneficial physiological functions, including immunoregulation, which facilitate energy metabolism and the remodeling of cardiac architecture, playing a role in improving insulin resistance and reducing systemic inflammation. Due to all these functions, sarcopenia has a systemic impact (Figure 2). Myostatin is a myokine that inhibits protein synthesis and regeneration; sarcopenia results from an imbalance between protein synthesis and catabolism [100].

Hyperammonemia plays a critical role in the development of sarcopenia, regulates the rise of myostatin levels, and activates autophagia; elevated ammonia levels lead to the impairment of the mTOR pathway through increased mitochondrial dysfunction and ROS production. Similarly, hyperammonemia affects muscle contractility and increases fatigue, contributing to muscle dysfunction [101].

**Figure 3:**
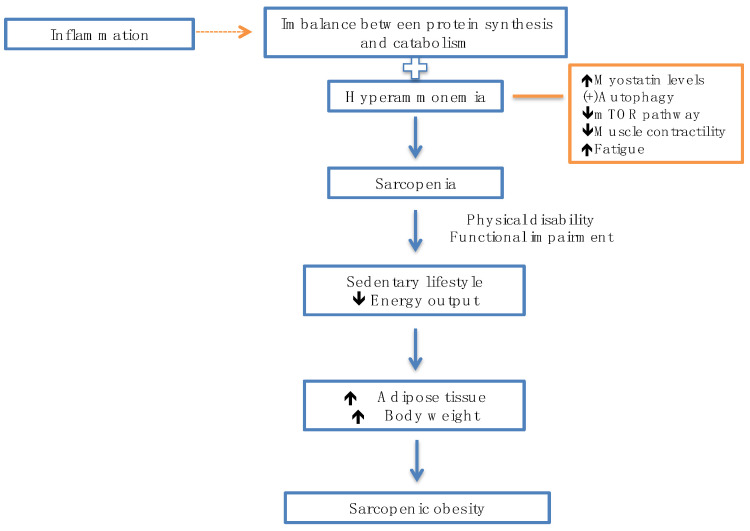
Inflammation and hyperammonemia predispose to the development of sarcopenia.

Sarcopenia conditions a sedentary lifestyle as a consequence of physical limitation and functional deterioration. Patients change their lifestyle, which, together with the decrease in energy expenditure, favors an increase in adipose tissue and obesity, leading to sarcopenic obesity; this increase in adipose tissue observed in sarcopenia activates SI [102] (Figure 3).

Patients are at risk of decompensation of liver function through the development of sarcopenia; this is associated with higher mortality, where the risk is five times higher. Meanwhile, the risk of developing cancer is also doubled, with a higher risk of infection [103].

## 2. Conclusions

Patients with liver cirrhosis present alterations mainly in the response of the innate immune system; however, the humoral immune system is also affected, which conditions an altered response to the recognition of pathogens. The liver is of great importance, as APCs are responsible for the immune response at local and systemic levels and as hepatocytes are responsible for PAMP and DAMP surveillance. The evolution of decompensated liver cirrhosis is characterized by repetitive episodes of acute decompensation events, which favor the presence of ACLF. It has been shown that patients with liver cirrhosis present alterations in the activation of the immune system. Following the recognition of PAMPs and DAMPs by PRRs, the pro-inflammatory pathway is activated to restore homeostasis through releasing CRP and pro-inflammatory cytokines. Patients with cirrhosis present an increase in intestinal permeability and a decrease in intestinal motility; both situations favor bacterial translocation, and these bacteria will also enter the systemic circulation. This explanation has been given to explain why patients with cirrhosis present constant immune system activation (low-grade inflammation). It is well known that bacterial infections are the triggers of ACLF. These types of patients have a higher risk of infection, which increases mortality at 3 months and 1 year. Furthermore, those who present with gastrointestinal bleeding have an increased risk of developing an infection. For this reason, there is an indication for the use of prophylactic antibiotics in patients with cirrhosis who are hospitalized for gastrointestinal bleeding. The use of antibiotics modifies the microbiota, thereby reducing the activation of immune cells in the mucosa which, in turn, reduces intestinal permeability and bacterial transfer. The presence of SBP confers a high rate of mortality and kidney damage. Hence, administering treatment based on intravenous albumin and antibiotics improves survival and reduces the risk of kidney damage. Within the group of patients with liver cirrhosis, the prognosis is also different: those who develop ACLF have a poor prognosis in the short and medium term, compared to both patients with acute decompensation without developing ACLF and patients with stable decompensated cirrhosis. Pro-inflammatory cytokines stimulate the endothelium to produce nitric oxide and ROS that favor the persistence of peripheral vasodilation, and inflammatory biomarkers change as liver function changes. Patients with ACLF present a high degree of systemic inflammation, evidenced by a decrease in TNF levels and an increase in IL-10. The presence of abnormalities in the cells of the immune system compromises the function of the cells and causes immunological paralysis. Both immunodeficiency and persistent systemic inflammation are characteristic of cirrhosis-associated immune dysfunction (CAID), affecting both innate and adaptive immune cells. Immunodeficiency is marked by an alteration in APCs, a decrease in HLA-DR expression, and an alteration in the phagocytic capacity of the cells. Another factor involved in the risk of developing infections is hypoalbuminemia, as albumin binds to pro-inflammatory cytokines and immunosuppression mediators. GALT is the first defense against pathogens. Moreover, the liver–intestinal axis plays an important role in the pathogenesis of CAID. The presence of SIRS can precipitate the presence of varicella hemorrhage and encephalopathy, worsening the prognosis. The presence of sarcopenia is a frequent complication in liver cirrhosis and negatively affects the health of these patients, deteriorates their quality of life, and worsens their prognosis due to the beneficial physiological effects of skeletal muscle as a regulator of the immune system. The development of hepatic encephalopathy is associated with a poor prognosis. In both HE and sarcopenia, hyperammonemia has a deleterious function, as it can perpetuate the inflammatory state.

## Figures and Tables

**Table 1:** Damage to the immune cells in cirrhosis [3].

Immune Cells	Decompensated Cirrhosis	ACLF
Monocytes	⇑ HLA-DR expression ⇑ TNF production ⇑ CD14+ CD16+ ⇓ Capacity of the APC Altered phagocytes Altered chemotaxis Production of OH^+^ Defective production of Fc	⇓ HLA-DR ⇑ Production of IL-10 ⇓ Production of TNF Altered phagocytosis ⇑ MERKT inhibits the activity of TLR, and the pro-inflammatory cytokines inhibit the activity of NF-kB
Neutrophils	Altered phagocytosis Reduced chemotaxis ⇑ ROS	⇑ Expression of CXCR1 and CXCR2 that favor apoptosis and necrosis
Lymphocytes	TCD4+ ⇓ Cytolytic activity of the NK cells	

## Data Availability

Data are contained within this article.
